# Oncologic Outcomes after Percutaneous Ablation for Colorectal Liver Metastases: An Updated Comprehensive Review

**DOI:** 10.3390/medicina60091536

**Published:** 2024-09-20

**Authors:** David-Dimitris Chlorogiannis, Vlasios S. Sotirchos, Constantinos T. Sofocleous

**Affiliations:** 1 Department of Radiology, Brigham and Women’s Hospital, Harvard Medical School, Boston, MA 02215, USA; dchlorogiannis@bwh.harvard.edu; 2 Interventional Oncology/IR Service, Department of Radiology, Memorial Sloan Kettering Cancer Center, New York, NY 10065, USA

**Keywords:** colorectal cancer, liver metastases, ablation margin, tumor ablation, interventional oncology, irreversible electroporation, histotripsy, locoregional therapy, hepatic malignancy

## Abstract

**Simple Summary:**

Colorectal cancer (CRC) remains a leading cancer-related mortality cause. More than a third of CRC patients develop liver metastases during the course of the disease, contributing to poor prognosis. Thermal ablation techniques are currently recommended as a local curative option for selected small liver metastases. Thermal ablation (TA) includes a wide variety of different modalities, like radiofrequency ablation (RFA), microwave ablation (MWA) and cryoablation (CRA). This comprehensive review outlines the role of image-guided ablation in the treatment of colorectal liver metastatic (CLM) disease.

**Abstract:**

Colorectal cancer is a major cause of cancer-related mortality, with liver metastases occurring in over a third of patients, and is correlated with poor prognosis. Despite surgical resection being the primary treatment option, only about 20% of patients qualify for surgery. Current guidelines recommend thermal ablation either alone or combined with surgery to treat limited hepatic metastases, provided that all visible disease can be effectively eradicated. Several ablation modalities, including radiofrequency ablation, microwave ablation, cryoablation, irreversible electroporation and histotripsy, are part of the percutaneous ablation armamentarium. Thermal ablation, including radiofrequency, microwave ablation and cryoablation, can offer local tumor control rates comparable to limited resection for selected tumors that can be ablated with margins. This review aims to encapsulate the current clinical evidence regarding the efficacy and oncologic outcomes after percutaneous ablation for the treatment of colorectal liver metastatic disease.

## 1. Introduction

Colorectal cancer (CRC) is the third most frequently diagnosed cancer and the second leading cause of cancer-related mortality in the United States [1]. Despite refinements in screening, the identification of preventable risk factors and treatment, recent population-based evidence highlights an alarming trend in the incidence of CRC in younger populations (<50 years) [1,2]. Moreover, over the course of the disease, 30–50% of CRC patients will develop liver involvement, which correlates with poor prognosis [3]. Hepatectomy has traditionally been used to provide a local cure for colorectal liver metastases (CLMs) in eligible patients. Nonetheless, approximately 20% of CLM patients qualify for surgery. Percutaneous thermal ablation is a minimally invasive curative-intent treatment option that has been proposed either alone or in combination with hepatectomy for local tumor control. According to the National Comprehensive Cancer Network guidelines (NCCN), percutaneous thermal ablation is recommended either alone or in combination with surgery, provided that all visible disease can be ablated with clear margins [4,5].

Percutaneous thermal ablation modalities involve the local deposition of thermal energy in the form of lethal heat, like radiofrequency ablation (RFA), microwave ablation (MWA), or lethal freezing with cryoablation (CA). Non-thermal ablative technologies include irreversible electroporation (IRE) and histotripsy. These techniques aim for tumor cell destruction either by exposing the tissue to heating (RFA, MWA) or freezing temperatures (CA), while IRE utilizes electromagnetic energy that causes tissue necrosis through irreversible cell membrane permeabilization, providing excellent oncologic outcomes [6,7,8]. Recent preliminary evidence points to the use of image-guided acoustic energy (histotripsy) as another alternative non-thermal ablative option in selected patients; especially those with tumors in locations considered at high risk for thermal ablation. There is also an evolving role of Y90 radiation segmentectomy as an ablative therapy that is beyond the scope of this review. Similarly, image-guided radiation therapy can offer ablative doses in cases that are deemed non-eligible for thermal ablation. 

This comprehensive review aims to encapsulate the contemporary clinical evidence regarding the oncologic outcomes of image-guided percutaneous thermal and non-thermal ablation techniques as a local therapy for CLM. 

## 2. Percutaneous Ablation Modalities

### 2.1. Radiofrequency Ablation

RFA has been extensively studied as a percutaneous local treatment for CLM and is considered the prototypical thermal ablation technique. The principle behind RFA is the induction of targeted thermal damage through the delivery of high-frequency alternating electric currents (365–500 kHz) via a monopolar or bipolar needle/electrode [9,10]. This effect induces ionic oscillation and frictional heating, leading to coagulation necrosis within the target tumor and surrounding tissue (margin). When tissue temperatures exceed 50 °C, protein denaturation occurs, causing cytotoxicity within 5 min, while at 60–100 °C, irreversible protein coagulation with subsequent coagulative necrosis takes place. After temperature reaches >100 °C, tissue desiccation and charring occur due to water evaporation, resulting in increased tissue impedance. This limits the volume of thermal energy transmission, preventing the lethal temperature from being delivered in the entire tumor and extending to surrounding tissues, resulting in a suboptimal treatment effect [11,12]. Apart from tissue desiccation, which is a technical limitation of RFA, another important limitation is the “heat-sink” effect occurring when the target tumor is proximal (within 1 cm) to a blood vessel 3 mm or larger, where the flowing blood interferes with energy deposition due to heat loss through the flowing blood and away from the target tumor. This effect attenuates cell death and thus increases the risk for local tumor progression [12,13]. Other known limitations of RFA include its relative increased procedure time, the inconsistency of obtaining large ablation margins and the less-substantiated concerns for needle tract seeding [14].

There is a great wealth of evidence regarding the survival outcomes after RFA for the treatment of patients with CLMs. Early results demonstrated that RFA was an effective local therapy for recurrences post-hepatectomy [15,16]. In more detail, several observational studies have reported long-term data with survival rates of 93%, 69%, 35% and 18% at 1, 3, 5 and 10 years, respectively [17,18]. Similarly, an observational study that included 162 patients with 233 CLMs treated with RFA reported an overall survival (OS) rate in 1 year, 3 years and 5 years of 90%, 48% and 31%, respectively, while the median local tumor progression-free survival (LTPFS) was 26 months [4]. In line with these results, a multicenter cohort that included a total of 238 patients with 496 CLMs treated with either CT- or MRI-RFA reported 1-, 5- and 10-year survival rates of 85.9%, 25.5% and 19.1%, respectively [19].

The efficacy of RFA with systemic chemotherapy was examined in a randomized controlled study (RCT), in which 119 patients with CLMs were randomized for systemic oxaliplatin-based chemotherapy alone vs. the same chemotherapy and RFA [20]. At a median follow-up of 9.7 years, the RFA group demonstrated a statistically significant prolongation of OS as compared to the systemic treatment arm (HR: 0.58, 95%; CI: 0.38–0.88, *p*-value < 0.05). In more detail, the 3-, 5- and 8-year OS rates in the systemic treatment arm were 55.2%, 30.3% and 8.9% vs. 56.9%, 43.1% and 35.9% in the RFA arm. Similar results were found for progression-free survival (PFS), which was improved from 9.9 in the chemotherapy-only arm to 16.8 months with the addition of RFA. A limitation of this study was the confounding of the RFA effects, since approximately half of these patients also received liver resection. However, the robustness of the 8-year OS (8.9 vs. 35.9%) and 8-year DFS (22.3 vs. 2.0%) provides level I evidence that the eradication of all macroscopically visible CLMs with locoregional therapies is correlated with better oncologic outcomes and especially OS [7]. The comparison of RFA with partial hepatectomy has been evaluated, with a systematic review and meta-analysis comprising 22 studies and a total of 4385 patients with CLMs revealing that the pooled 1-, 3- and 5-year OS rates after RFA were lower than those of hepatic resection with pooled Odds Ratios (ORs) of 0.39, 0.40 and 0.60, respectively [21]. A lower 5-year disease-free survival (DFS) rate was also found higher in the RFA than in the hepatic resection group, with a pooled OR of 0.74 (95% CI: 0.56–0.97; *p*-value < 0.05). Of note, the included studies lacked stratification of outcomes with the ability to treat with optimal ablation margins, while the increased heterogeneity of the included studies and population characteristics make the generalization of these results challenging [21,22]. 

The increasing evidence regarding factors associated with better outcomes after percutaneous image-guided thermal ablation has optimized individualized care for metastatic colorectal disease. Many studies have attributed increased risk for LTP to the tumor mutation status, tumor size, proximity to large vessels and subcapsular tumor location [23,24]. Nonetheless, minimal ablation margins and tumor size have been identified as the most robust predictors for LTP in multiple large observational studies [6,25,26,27,28]. Moreover, as recommended by a panel of experts, the ultimate treatment goal after percutaneous ablation is an ablation margin of at least 10 mm [29]. A recent systematic review and meta-analysis that evaluated aggregated data from 21 studies provided conclusive evidence on the superiority of a 1 cm minimal ablation margin (MM) and reported an 8.31-times higher risk for LTP when the MM was <10 mm vs. ≥10 mm (RR: 8.31; 95% CI: 3.38–20.43; *p*-value < 0.001) [30]. Nonetheless, despite its ability to provide oncologic results comparable to surgery, the achievement of an ablation margin of 10 mm is challenging and may be associated with increased liver adverse events in selected high-risk patients, especially those with prior exposure to hepatic arterial chemotherapy, bevacuzimab and pre-existing biliary dilatation [6]. In this context, prospective phase II studies reported that concomitant negative biopsy findings of the ablation zone and ablation margins ≥ 5 mm is associated with a cumulative 2-year LTP rate of 3 to 7%, highlighting the potential usage of biopsy confirmation of complete ablation especially in cases where a larger ablation margin cannot be obtained [31,32]. Similarly, an increased tumor size undermines the efficacy of thermal ablation regardless of ablation modality, as reported in large retrospective studies. In a retrospective study, LTP after RFA was 9%, 26.5% and 45% for CLMs of 0–3 cm, 3–5 cm and >5 cm, respectively [33]. By evaluating data from the Amsterdam Colorectal Liver Met Registry, Dijkstra et al. found an overall higher LTP and higher LTPFS rates between intermediate vs. smaller tumors after thermal ablation (HR: 3.7 and HR: 3.39; *p*-value < 0.001, respectively) with no difference in the complication rate and 5-year OS [34]. However, evidence regarding the optimal technique for intermediate-sized CLMs is lacking, mainly due to the increased heterogeneity between studies within the existing literature and no direct comparison between techniques [35].

### 2.2. Microwave Ablation

**Figure 1:**
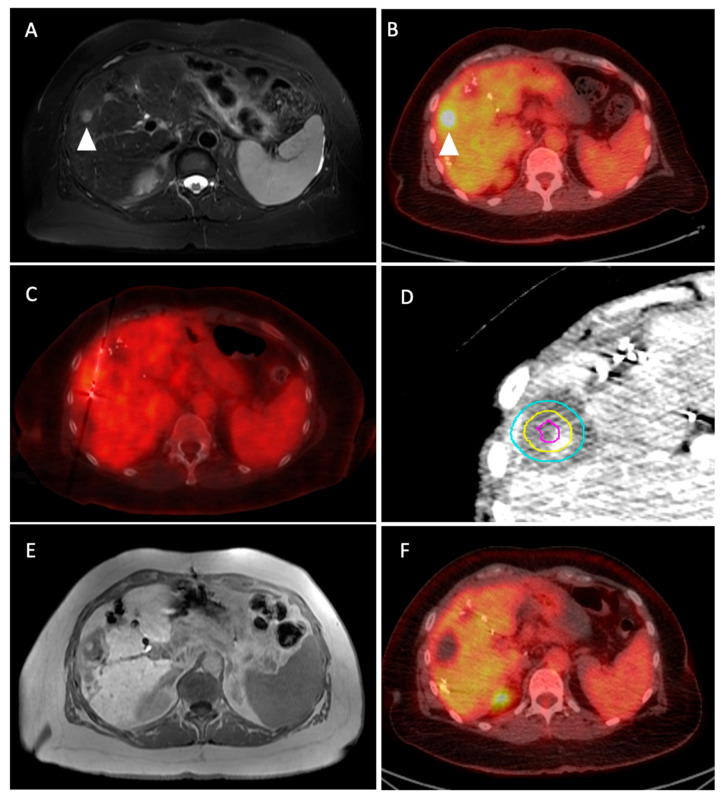
Sixty-one-year-old woman with metastatic colon cancer, status post chemotherapy, partial colectomy, hepatic arterial infusion pump chemotherapy and hepatic resection with intra-operative ablations, with new 1.2 cm segment 5 T2 hyperintense ((**A**): arrowhead)) FDG-avid (**B**) metastasis. This was treated with PET/CT-guided microwave ablation (**C**). The tumor was segmented on the immediate pre-ablation portal venous-phase CT, and subsequently registered to the post-ablation portal venous-phase CT ((**D**); purple border: segmented tumor, yellow border: 5 mm margin, light blue border: 10 mm margin), showing coverage of the tumor with adequate margins. Follow-up imaging after 6 weeks shows the segment 5 ablation zone without evidence of residual tumor ((**E**): hepatobiliary phase of MRI), with corresponding photopenic defect on concurrent PET/CT (**F**).

MWA utilizes electromagnetic energy operating at frequencies between 900 MHz and 2.45 GHz. Polar molecules in the tissue (mainly H_2_O) are forced to rotate and realign continuously with the electric field, leading to frictional heating and subsequent protein denaturation with coagulative necrosis [36,37]. MWA, unlike RFA, does not require grounding pads and can operate multiple antennae simultaneously, thus increasing ablation volumes, within a shorter period of time. Additionally, MWA is less affected by the low thermal conductivity of surrounding and charred tissues, enabling the effective treatment of larger tumors, including tumors located near large vessels, making it less susceptible to impedance and heat-sink effects [38,39,40,41] (Figure 1). 

Regarding the efficacy of MWA over RFA for the treatment of liver metastases including CRC (MWA: 13, RFA: 14), one trial randomized 50 patients to either treatment, with a follow-up of 2 years [42]. Even though there was 0% LTP in the MWA group and 8.3% in the RFA group, this difference was not statistically significant (*p*-value > 0.05). Additionally, the investigators reported that there were no differences in overall survival, intrahepatic progression and complications between the two thermal ablation modalities [42]. This result was also highlighted by a systematic review and meta-analysis by Di Martino et al., which reported the aggregated rates of RFA and MWA with respect to adverse events (8% RFA; 7% MWA), 5-year DFS (18% RFA; 38.5% MWA) and 5-year OS (43% RFA; 55% MWA) [43]. However, these data stemmed from mainly single-arm studies, and thus cannot be directly compared. Confirming these findings, a systematic review evaluated aggregated data from five studies (MWA: 316 patients; and RFA: 332 patients) [44]. The authors concluded that the odds for LTP in the RFA group were significantly higher than in the group that underwent MWA (OR: 3.03; 95% CI: 1.59–5.79; *p*-value < 0.05), while the pooled risk for 1-year and 2-year DFS was also significantly higher for RFA vs. MWA ((HR: 1.77; 95% CI: 1.04–3.02; *p*-value < 0.05) and (HR: 1.60; 95% CI: 1.09–2.35 *p*-value < 0.05), respectively). It should be noted, however, that none of these results were stratified by margins. In this matter, a retrospective study that compared the efficacy of RFA vs. MWA after ablation margin stratification reported no difference in the LTP rates between the two (3 vs. 7%; *p*-value > 0.05) with the 2-year cumulative LTP rates being 36% and 38%, respectively (*p*-value > 0.05) [28]. 

In patients with CLMs, the comparison of MWA to partial hepatectomy, considered the standard of care for resectable diseases, has also been a subject of extensive research. Regarding high-quality evidence from RCTs, the multicenter LAVA trial, which ended abruptly, highlighted the challenges in recruitment and potential subtle selection bias favoring surgery [45]. The COLLISION trial recently reported Level IB evidence by announcing its final results during the American Society of Clinical Oncology 2024 annual meeting [46]. This multicenter phase 3 trial randomized patients to either surgery (n = 149) or thermal ablation (n = 147). Inclusion criteria were previously untreated ≤10 CLMs up to 3 cm in size, no extrahepatic metastases and ECOG status of 0–2. After a median follow-up of 29 months, the investigators found that thermal ablation was non-inferior to surgery regarding OS (HR 1; 95% CI 0.7–1.6; *p*-value > 0.05). Additionally, thermal ablation was not statistically different regarding LTPFS (HR 0.8; 95% CI 0.5–1.5; *p*-value > 0.05) and distant PFS (HR 1.0; 95% CI 0.7–1.3; *p*-value > 0.05), while local control favored thermal ablation (HR 0.18; 95% CI, 0.04–0.84; *p*-value > 0.05). No procedure-related mortality was observed for thermal ablation, while it was 2.1% (n = 3) for resection, solidifying the excellent safety profile of thermal ablation. Several ongoing RCTs are also designed to compare the efficacy of thermal ablation to partial hepatectomy. These include the NEW-COMET trial (NCT05129787) and the HELARC trial (NCT02886104), both of which are expected to be completed in 2026. In this domain, the ongoing prospective CIRSE Emprint Microwave Ablation Registry (CIEMAR; NCT03775980) also aims to provide real-world data on the usage of MWA for the treatment of patients with CLMs with a focus on 1-year LTP rates and quality of life outcomes [47], expected to also be completed by 2026. Additionally, the Ablation with Confirmation of Colorectal Liver Metastases trial (ACCLAIM) aims to provide definite evidence on the efficacy of MWA as a local cure for the treatment of up to three liver metastases (each ≤2.5 cm) when minimal ablation margins are confirmed to be ≥5 mm, with an estimate completion date of 2027 (NCT05265169, 13 January 2023). Prospective data evaluating the non-inferiority of stereotactic microwave ablation compared to surgical resection are available from the MAVERICC trial [48]. In this study, 98 patients who underwent stereotactic microwave ablation were matched with 158 patients from the surgical group, revealing comparable 5-year overall survival rates (56%, 95% CI: 45–66% vs. 58%, 95% CI: 50–66%) and significantly lower overall and major complication rates in the ablation group (percentage decrease of 67% and 80%, *p*-value < 0.05). Several observational retrospective studies have also provided real-world data concerning the long-term efficacy of MWA [6,34,49,50]. A retrospective monocentric study included 2140 patients with CLMs, treated using various locoregional therapies, including thermal ablation with MWA, transarterial chemoembolization (TACE), laser-induced thermotherapy (LITT) and combinations, in a span of 26 years [50]. The authors highlighted that the longest survival benefit was found for the MWA group when compared to other locoregional techniques with a mean OS of 7.6 years in the MWA group and 1-, 3- and 5-year OS rates of 80%, 55% and 33%, respectively. Lastly, by using data from the Amsterdam Colorectal Liver met Registry (AmCORE), Dijkstra et al. provided evidence regarding the efficacy of thermal ablation for the treatment of patients with recurrent CLMs vs. repeat partial hepatectomy. The study reported no difference in the crude and adjusted OS, LTPFS or disease progression-free survival between the two techniques, further highlighting the increasing role of thermal ablation even in this patient population [51]. 

### 2.3. Cryoablation

Cryoablation is a technique that utilizes the Joules–Thomson effect by administering liquid nitrogen or argon via a needle probe to rapidly decrease tissue temperature until it reaches lethal levels (−20 to −40 °C). The rapid freezing first forms extracellular ice, causing a hyperosmotic state that draws the intracellular free water and subsequently damages the cell and, as temperatures drop, the intracellular ice that forms further injures the cell membranes and organelles. This is followed by a slow thaw cycle, in which ice melts and water enters the cell, causing osmotic expansion, disruption to the cell membrane and apoptosis [45,46]. Tissue temperature differs in different areas of the ice-ball, with the temperature at the periphery being 0 °C (non-lethal), while 5 mm inward, the temperature is approximately −20 °C. As with other ablation modalities, the goal is to cover the lesion while also creating the necessary ablation margins (A0), and thus this ablation modality usually involves inserting one or more cryoprobes into or around the target tumor. A conferred benefit of cryoablation is the intra-procedural visualization of the ice-ball, either with ultrasound, CT or MRI, although this advantage has often been met with skepticism, with newer studies revealing that the ice-ball may overestimate the true lethal ablation zone [47,48]. 

Even though cryoablation was one of the first ablation techniques used for liver metastases, its widespread adoption was limited due to preliminary data of high LTP rates and increased incidences of “cryo-shock” syndrome, characterized by multiorgan failure from disseminated intravascular coagulation and renal and respiratory failure [49,50,51]. However, these historic concerns are being overwrought as newer data come to the surface, especially for tumors < 4 cm in size and tumors adjacent to critical structures such as vessels and central bile ducts [8,52]. Evidence regarding the efficacy of cryoablation for liver metastasis was evaluated in a systematic review and meta-analysis that included 15 studies [53]. The authors reported a very high range in both the incidence of LTP (9.4–78%) and mean overall survival (14.5–29 months); however, they noted an increase in the quality of life after one month post-procedure (5.69; 95% CI: 3.99–7.39, *p*-value < 0.05). In a similar context, a different meta-analysis that aimed to compare different thermal ablation therapies for resectable CLM disease by pooling data from 20 studies showed a similar adverse event rate between RFA, MWA and cryoablation, but the median 3-year OS was the lowest for the latter (60% RFA; 70% MWA and 34% for cryoablation) [43]. The external validity for these two studies was limited by the inherent variability among the baseline characteristics of the population, like cancer origin, extent of disease and not stratifying by margins. Lastly, early to intermediate term survival outcomes were reported in two large retrospective series that evaluated the efficacy of percutaneous cryoablation for liver metastases. At a mean follow-up time of 1.8 and 2.5 years, LTP rates were 11.1 and 24.6%, respectively. 

### 2.4. Irreversible Electroporation

Irreversible electroporation (IRE) is the most used non-thermal ablation technique. IRE uses high-voltage pulses to cause cellular membrane disruption leading to cell death. In more detail, this effect is achieved by the permeabilization of the lipid cellular membrane after ultra-short pulses of a high electric current, creating permanent (irreversible) nanopores [54,55]. These nanopores alter cellular homeostasis, promoting osmotic lysis, apoptosis and ultimately cell death [56]. Even though a small amount of heat is also generated, the temperatures do not reach lethal levels; thus, surrounding connective tissue architecture is preserved [57]. Hence, IRE is primarily applied for the treatment of tumors in challenging locations such as proximal to anatomic structures like blood vessels, bile ducts or the liver hilum [58,59]. IRE needs to be performed under general anesthesia, complete muscle relaxation and simultaneous electrocardiogram synchronization to prevent unintended generalized muscle contractions and arrythmia caused by its high-voltage pulses [60]. 

The efficacy of IRE to eradicate colorectal liver metastases was demonstrated in the COLDFIRE-1-ablate-and-resect study and the subsequent COLDFIRE-2 single-arm clinical trial that included 51 patients with 76 CLMs unsuitable for surgery or thermal ablation [61,62]. The investigators reported a per-tumor 1-year LTP-free survival rate of 79%, median DFS of 5.3 months and median OS of 2.7 years, with an overall complication rate of 40% (25/62 procedures), with the most severe being cardiac arrhythmia (n = 4), portal vein thrombosis (n = 3) and biliary obstruction (n = 3), though none of which were Grade 4 or 5. A subsequent systematic review of eight studies involving the efficacy and safety of IRE for the treatment of 283 tumors in 162 patients revealed that LTPFS ranged from 0 (for incompletely treated disease) to 10 months, while the mean OS was 61.5% at 2 years [63]. Regarding long-term outcomes, a nationwide multicenter trial reported its results for hepatocellular carcinoma and CLM disease treated with IRE [64]. From a total of 87 patients, the median time to LTP for patients with CLM was 6.0 months (95% CI: 4.5–7.5 months). At a 30-day follow-up, 15.4% of patients of the full cohort suffered from a complication rated as Clavien–Dindo grade 1–3A. Of note, after a Cox regression model analysis, a tumor size of ≥3 cm was found as a negative predictor for OS in the CLM cohort (HR 3.24 95% CI 1.43–7.33, *p*-value < 0.05). A systematic review and meta-analysis that aimed to compare the efficacy between different thermal ablation techniques for an intermediate 3–5 cm tumor size revealed that the per-patient local tumor control ranged 22–89% for any thermal ablation technique, but was approximately 44% for IRE, though this comparison cannot be made with certainty due to the heterogeneity of the included population [35]. Lastly, in line with this evidence, a recent retrospective study evaluated the efficacy of IRE for the treatment of CLM in proximity to key structures. This study included 26 patients with 46 tumors in challenging locations and reported 1- and 2-year LTPFS of 55.0% and 51.3%, while the 1- and 2-year OS was 90.9% and 83.9% [65]. 

### 2.5. Histotripsy 

Histotripsy is a novel non-invasive, non-thermal and non-ionizing energy therapeutic modality that has the additional benefit of real-time visualization of the tissue effect. The fundamental process behind histotripsy is the controlled generation of acoustic cavitation and its interaction with tissue, resulting in tissue breakdown [66]. This is caused by the interaction between the acoustic waves and nanometer-scale gas pockets within tissues, acting as nucleation sites for cavitation [67]. With sufficiently high pressure and an adequate number of pulses, the target tissue can be fully destroyed, resulting in a fluid homogenate. Early preclinical studies confirmed its effectiveness in obliterating soft tissues in large animal models with precision at the tissue level and direct sonographic visualization [68,69]. These promising results fueled the research interest for human clinical studies. As such, the THERESA trial evaluated histotripsy in eight patients with eleven liver malignancies, including seven CLMs [70]. The authors reported 100% technical success and no device-related adverse events in a 2-month follow up. The percent of residual tumors 1 month after treatment was 20%. Recently, the early results of the prospective multicenter single arm HOPE4LIVER trial were published [71]. This trial evaluated 44 patients with 49 liver tumors treated with histotripsy. All tumors were ≤3 cm large in diameter. From the 44 patients, 10 were treated for colorectal liver metastases. The investigators reported the achievement of complete tumor coverage in 42 tumors (95%) while also reporting a 7% (3 of 44) procedure-related major complication rate (Common Terminology Criteria for Adverse Events ≥ 3) within 30 days post-procedure, highlighting its safety and clinical efficacy. Further results regarding adverse events and oncologic outcomes after 6 months of follow-up are also expected from the same investigator group. The completion date is expected to be 2026 (https://clinicaltrials.gov/study/NCT04573881 (accessed on 20 July 2024)) [72]. 

## 3. Discussion

Percutaneous thermal ablation has revolutionized the treatment of colorectal liver metastatic disease with multiple ablation modalities constituting the interventional radiologist’s therapeutic armamentarium. Overall, RFA has been meticulously studied for decades and is readily available as a cost-efficient modality with proven safety and oncologic efficacy. However, in the contemporary clinical setting, its usage has largely been replaced in favor of newer ablation systems due to its inherent limitations. These include its decreased energy disposition on the liver, smaller ablation volumes in comparison to MWA, vulnerability to the heat-sink effect and increased procedure time [73,74]. Indeed, the heat-sink effect negatively impacts the LTP rates after RFA [75]. This is especially true for perivascular tumors, where the LTP after RFA is higher compared to MWA, which exhibits resistance to this phenomenon, as shown in bioclinical and animal studies [28,44,74,76]. MWA allows for more energy disposition and is less affected by the conductivity of surrounding tissue, thus achieving higher temperatures faster than other techniques. This leads to larger ablation zones, especially when multiple needles are combined effectively, inducing coagulation necrosis in the targeted tissue [77]. As such, its enhanced efficacy makes it ideal for targeting tumors that can be ablated with clear margins [42,74]. 

In this domain, cryoablation’s biggest advantage over the other modalities is that it offers easier intra-operative monitoring of the ablation zone. The ice-ball formation is visible under both non-contrast-enhanced CT and MRI, with specifically designed antennas for both imaging modalities. Furthermore, another key advantage of cryoablation lies in the plethora of different probes that produce specific ice-ball shapes. As such, the utilization of multiple probes allows for the “sculpting of the ablation zone”, which is extremely important when treating tumors in close proximity to key anatomical structures such as the bile ducts, liver hilum, major vessels and the heart [78]. Nonetheless, these advantages come at the expense of increased cost and prolonged procedure time when compared to MWA, while its efficacy is also reduced for larger sized tumors (>4 cm) [6,8]. The typical double-freeze cycle requires a 15 min freeze–10 min thaw–15 min freeze cycle, totaling up to a 40 min procedure time. Additionally, it is not recommended in selected cases, especially in patients with a low platelet count, due to associated thrombocytopenia and bleeding [79]. Furthermore, early reports of cryoshock after cryoablation have limited its use for liver tumors [80]. 

Similarly, IRE is an important technique when treating tumors in challenging locations. IRE is completely independent of the heat-sink effect, is tumor selective and offers minimal risk for collateral damage to adjacent structures. However, major disadvantages include its relatively small ablation zone, the requirement of careful parallel needle placement, the need for general anesthesia with complete paralysis and the need for concomitant neuromuscular and cardiac monitoring. Additionally, the risk for arrhythmias, though rare, is not negligible. Of note, its efficacy for local tumor control, even at such locations, necessitates further research, since reports regarding its oncologic outcomes highlight that it falls short when compared to the established modalities of thermal ablation, including RFA and MWA [65]. Lastly, despite the promising early results of histotripsy as the first non-ionizing and non-invasive modality, further research is still necessary to overcome its limitations regarding its relatively small ablation zones, need for a proper acoustic window, high cost and limited availability of the equipment. Lastly, the evidence regarding its clinical efficacy with regard to long-term local tumor control and complications, as well as its potential immunomodulation effects, remains premature. Thus, histotripsy can only be considered when thermal ablation is deemed risky or not feasible or in the context of clinical trials.

## 4. Conclusions

Percutaneous ablation using thermal or non-thermal energy has revolutionized colorectal liver disease patient care and has been solidified as a primary curative-intent treatment option for patients with limited disease, especially after the results of large, randomized studies comparing it to surgery. Percutaneous ablation modalities continue to be refined, with data from ongoing studies being expected to play a pivotal role in the identification of the respective patient populations that will benefit the most from each respective modality, aiming towards optimal oncologic outcomes.
